# The interplay between cancer type, panel size and tumor mutational burden threshold in patient selection for cancer immunotherapy

**DOI:** 10.1371/journal.pcbi.1008332

**Published:** 2020-11-09

**Authors:** Mahdi Golkaram, Chen Zhao, Kristina Kruglyak, Shile Zhang, Sven Bilke

**Affiliations:** Illumina, Inc., San Diego, CA, United States of America; Institute for Disease Modeling, UNITED STATES

## Abstract

The tumor mutational burden (TMB) is increasingly recognized as an emerging biomarker that predicts improved outcomes or response to immune checkpoint inhibitors in cancer. A multitude of technical and biological factors make it difficult to compare TMB values across platforms, histologies, and treatments. Here, we present a mechanistic model that explains the association between panel size, histology, and TMB threshold with panel performance and survival outcome and demonstrate the limitations of existing methods utilized to harmonize TMB across platforms.

## Author summary

An increasing number of studies have demonstrated the benefit of tumor mutation burden (TMB), the number of non-silent mutations in the genome, as a predictive biomarker in a clinical setting. Most clinical trials utilize a smaller panel, instead of whole exome sequencing (WES), to estimate the exome-wide mutational load. However, the use of panels introduces panel size dependent sampling noise that could affect the performance of the TMB biomarker. In this work we create a mathematical model of the cancer histology, treatment response, and TMB device system to assess the interplay between cancer type, panel size and tumor mutational burden threshold in patient selection for cancer immunotherapy.

## Introduction

Recent trials have demonstrated the utility of TMB as a potential predictive biomarker in clinical settings [1–4]. Lung cancer patients with elevated TMB treated with Nivolumab and Ipilumab, for example, were found to have a 3-fold higher likelihood of one-year progression free survival compared to an unstratified control group receiving chemotherapy [1]. However, the question of what numerical TMB value constitutes an ‘elevated’ mutation burden turns out to be surprisingly complex. A multitude of factors including technical details of the TMB assay, pre-analytical choices, cancer tissue of origin, or the disease specific outcome achieved with standard of care inform this choice [5, 6]. Friends of Cancer Research (FRIENDS) and the Quality Assurance Initiative Pathology (QuIP) have initiated an international collaborative TMB harmonization effort based on both *in silico* and wet-lab experiments. These efforts led to critical insight [7] into how well distinct technical platforms compare. This work builds on these efforts by creating a mathematical framework that allows us to analyze how different technical factors interact. The mathematical framework presented here allows us to pinpoint certain technical limitations in the numerical methods currently used in harmonization efforts with the hope that these insights contribute to the development of improved pan-cancer algorithms for assay harmonization efforts.

Most clinical trials employ a panel design, instead of the “gold standard”, whole exome sequencing (WES), to estimate the exome-wide mutational load. The use of panels introduces panel size dependent sampling noise [8] that could affect the performance of the TMB biomarker: patients with a relatively low TMB may be incorrectly classified as high TMB and vice versa. But by how much? Could this be compensated by choosing a different threshold? We show that the answer depends not only on the amount of noise, but is intimately linked to other factors, such as the biology of the drug/ tumor interaction or the cancer tissue of origin (via the distribution of TMB in the intention to treat population).

## Results

Statistical mechanics were originally developed to provide a first-principle explanation of thermodynamics [9] but were soon applied in a diverse field of seemingly unrelated problems, including the strong interaction that keeps atomic nuclei together [10], quantitative stock analysis [11], hurricane prediction [12], and the dynamics of artificial neural networks [13]. In this work we borrow methods from statistical mechanics to create a mathematical model of the cancer histology, treatment response, and TMB device system. Within this framework, the individual components of the system are described as probability distributions, specifically, the histology- dependent distribution Π(*T*) models the probability that the cancer of a patient has a “true” or “noiseless” TMB (*T*). The assay may or may not correctly classify the patient as TMB high, and the probability distribution Θ(*T*,*τ*,*σ*) describes the resulting uncertainty that a TMB assay (with noise parameter *σ* and TMB threshold *τ*) classifies the patient as TMB high. Finally, response to treatment with checkpoint inhibitors (now on referred to as treatment), while dependent on TMB, is not guaranteed, and Ψ(*T*) expresses the probability that a patient with a “true” TMB of *T* will respond to treatment.

With these definitions it is now possible to estimate “observable” properties of the biomarker, such as *P*_*response*_(*τ*,*σ*), the fraction of cancer patients that respond to treatment (overall response rate or ORR) when stratified with a noisy biomarker:

$$P_{response}(\tau ,\sigma )=\frac{1}{Z(\tau ,\sigma )}\int dT\Pi (T)\Psi (T)\Theta (T,\tau ,\sigma ),$$ (1)

**Fig 1:**
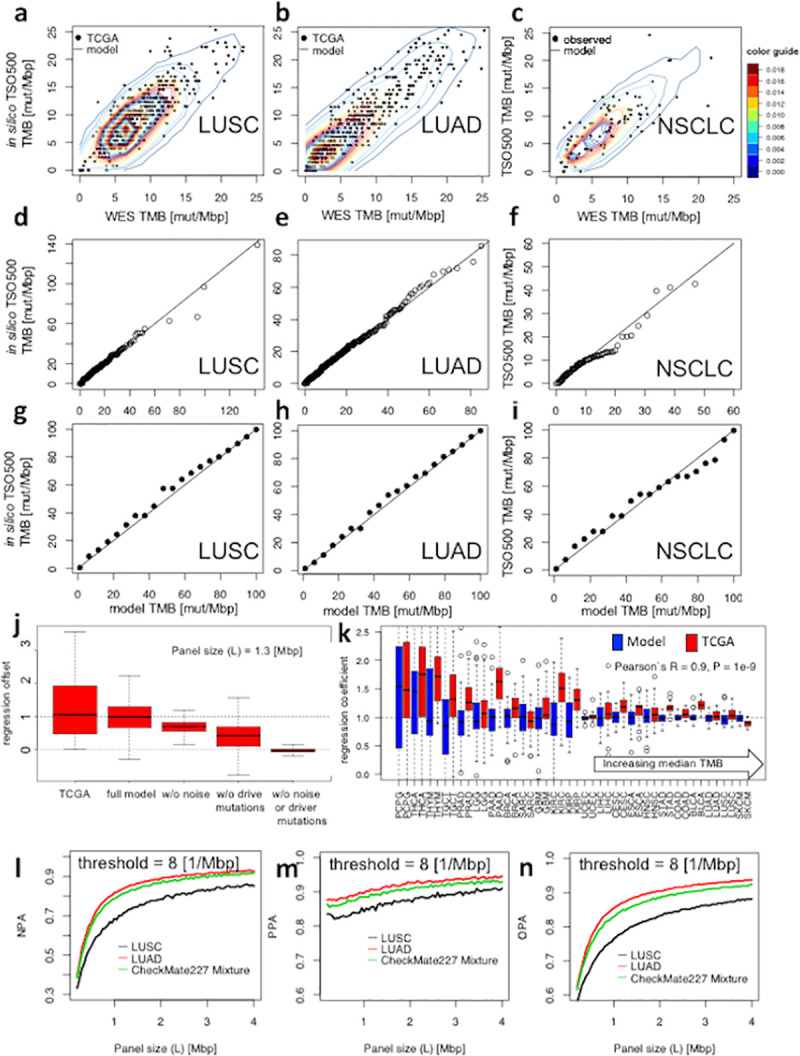
The model estimates TMB recordings by TSO500 panel and predicts the panel classification performance accurately. **a** and **b**, the model predictions are in agreement with training set (TCGA) for two subtypes of lung cancer. Data points represent TMB measurements using WES and the corresponding TMB values from *in silico* TSO500. Contours illustrate the predicted distributions of TMB by the model. Contour colors show the density of data points per contour. **d** and **e** show an agreement between the quantiles of recorded TMB values using *in silico* TSO500 and the predicted values by the model. **g** and **h**, comparison between the number of data points that lay within each percentile in **a** and **b** in TCGA and the model. **c,f,** and **i** show a striking agreement between the model predictions on test set (in-house generated TMB values using WES and TSO500 for NSCLC). **j**-**k**, linear regression relates TMB measurements differently for different panels and histologies. **j**, the model predicts the same regression offset as observed in TCGA by different sources of noise. **k,** regression coefficients depend on the histology as shown by both the model and TCGA. Histologies are ordered by increasing median TMB. Low TMB tissues in TCGA demonstrate a higher variability in regression slopes in agreement with the model predictions (Pearson`s R between the regression variability of TCGA and the model = 0.9, P = 1e-9). **l**-**n**, the model predicts the classification performance of different panels for different subtypes of lung cancer. NPA: negative percent agreement, PPA: positive percent agreement, OPA: overall percent agreement.

We first tested the accuracy of our panel size-dependent sampling noise model (see Methods). To this end, we predicted the outcome of the *in-silico* re-sampling experiment performed by FRIENDS [14]. That experiment utilized WES data from The Cancer Genome Atlas (TCGA) to generate *in silico* TMB measurements for a specific panel by intersecting WES reads with the targets of that panel (see Methods). We initially focused on validating our noise model underlying Θ(*T*,*τ*,*σ*) (see Methods) by comparing the TMB values generated with our content-agnostic, statistical model with the FRIENDS re-sampling method, hereinafter called *in silico* TruSight Oncology 500 (TSO500)–a research use assay. Utilizing the empirical WES distribution in TCGA as a starting point, we observed no significant difference between the observed distribution of TMB measurements reported by *in silico* TSO500 relative to WES and the distribution predicted by the model (Cramer test P = 0.88, and 0.77 for lung squamous cell carcinoma [LUSC] and lung adenocarcinoma [LUAD], Fig 1A and 1B). Likewise, comparing the quantiles as well as the density of data points belonging to each percentile demonstrated a high agreement between the predicted and observed data (Fig 1D, 1E, 1G and 1H).

Subsequently, we performed paired WES and panel (TSO500) sequencing on an independent cohort of NSCLC (which includes both LUSC and LUAD subtypes, n = 98, see Methods and S1 Table) to test the accuracy of our model. We determined the TMB of each tumor sample using both TSO500 and WES assays and observed a striking concordance between the TSO500 TMB measurements and the model predictions (Cramer test for difference: P = 0.4, Fig 1C, 1F and 1I). Moreover, our model was able to estimate and compare the noise content of panels of various sizes with high accuracy (S1A Fig). The comparison between the observed panel noise for this cohort and the theoretical noise content further validated the model’s accuracy (model *R*_*test*_^2^ = 0.9 vs. observed *R*_*test*_^2^ = 0.87).

One of the results of the FRIENDS analysis was an apparent histology dependence of how WES TMB values map to smaller panels [14]. This observation has a potential to complicate the development of diagnostic TMB applications as the expensive work of mapping of thresholds between assay platforms might have to be repeated for each histology. For pan-cancer applications, how should a common threshold be determined, and when mapping different assay, would it still be a single common threshold for all histologies? Our numerical model allows us to investigate this further. We used data synthesized by our statistical model that is explicitly designed to be histology agnostic. In our setup, the ground truth map is solely determined by panel size (*t*_*panel*_ ≔ *t*_*WES*_ . *L*_*panel*_/*L*_*WES*_) and therefore histology agnostic. With this setup, we synthesized panel TMB values from WES data and subsequently executed the same regression analysis that is typically performed for real world data. To our surprise, executing this procedure for 23 cancer types (TCGA data for cohorts larger than 100 subjects) quite closely reproduced the histology dependence of the regression coefficients found in the FRIENDS *in silico* panel mapping experiments (Fig 1J and 1K and S1B Fig). Given that the built-in ground truth in these experiments was histology independent, the counterintuitive observation of apparent histology dependence indicates that the method that is broadly used to map WES versus panel TMB is flawed. We speculate that the non-symmetric nature of small TMB values (negative TMB values are forbidden) is one contributing factor. Indeed, Fig 1K shows a higher variability of regression coefficients for tissue types with lower TMB as suggested by the model in agreement with TCGA observations. For further exploration, we next reduced various noise sources (e.g. sampling noise, driver mutations, germline mutations, etc., see Methods), and eventually achieved a tissue-independent mapping from TMB measurements of a panel to WES (no offset; Fig 1J). An additional factor may be the discrete nature of TMB, in particular on smaller panels that makes the noise distribution very non-normal. The tissue type dependency of regression coefficients can also be seen when TMB values from a panel (size 1 Mbp) are mapped to a larger panel (size 2 Mbp) (S1C Fig). Likewise, the model demonstrated that this effect is accentuated in smaller panels (i.e. higher noise content) (S1B Fig).

Earlier studies demonstrated a promising association between TMB and response to treatment with immune checkpoint inhibitors [3, 15, 16]. But subsequent prospective trials stratifying patients in TMB low and high groups, while generally confirmatory, did not always meet the high expectations of TMB as a clinical biomarker as demonstrated in KEYNOTE-021 C and G (nonsquamous; NCT02039674), 189 (nonsquamous; NCT02578680), and 407 (squamous; NCT02775435) [17]. In practice it would be difficult to delineate experimentally how much purely technical factors may affect the predictive performance of TMB, as opposed to intrinsic limitations of the biomarker itself [18]. With the help of the mathematical model presented here (Eq 1), it is straightforward to approximate how different factors such as panel size, threshold, and cancer type can affect the sensitivity and specificity of TMB classification. Initially, we focused on NSCLC as an example. As intuitively expected, we found that the panel size affected classification accuracy. Classification accuracy also depends on cancer type. Namely, LUSC subtype tends to render less accurate classification compared to LUAD originating from different TMB distributions. We hypothesized that this may be due to the fact that LUSC subtype is enriched for smokers who have a tendency to present with a higher number of mutations, while LUAD subtype displays a long tail distribution facilitating easier classification (as seen in TMB distributions of LUAD and LUSC cohorts of TCGA). Similar analysis can guide drug/companion diagnostics (CDx) co-developers to accurately account for the impact of heterogeneity of distinct cohorts in clinical trials (e.g. CheckMate227 [1], Fig 1L–1N, and S2A–S2O Fig).

Furthermore, our analysis demonstrates the threshold selection not only affects classification accuracy, but it also accentuates how strongly performance depends on panel size (S2A–S2O Fig). Tissue type is another factor that can influence classification accuracy. For example, even assuming a tissue agnostic threshold, classification accuracy of TMB using the same panel strongly depends on tissue type due to distinct TMB distribution of each particular histology. Notably, the model illustrates that histologies with low TMB (e.g. prostate adenocarcinoma, PRAD or breast cancer, BRCA) suffer from low positive predictive value (PPV) while leading to superior overall predictive agreement (OPA) compared to histologies with high TMB. This observation is even more pronounced when smaller panels are used, suggesting the value of comparatively larger panels such as TSO500 and WES for future clinical trials in prostate, breast cancer or other histologies with low TMB (S2A–S2O Fig). Our model concludes that the performance of panels plateaus around ~1 Mbp for most thresholds and cancer types in agreement with others [19].

**Fig 2:**
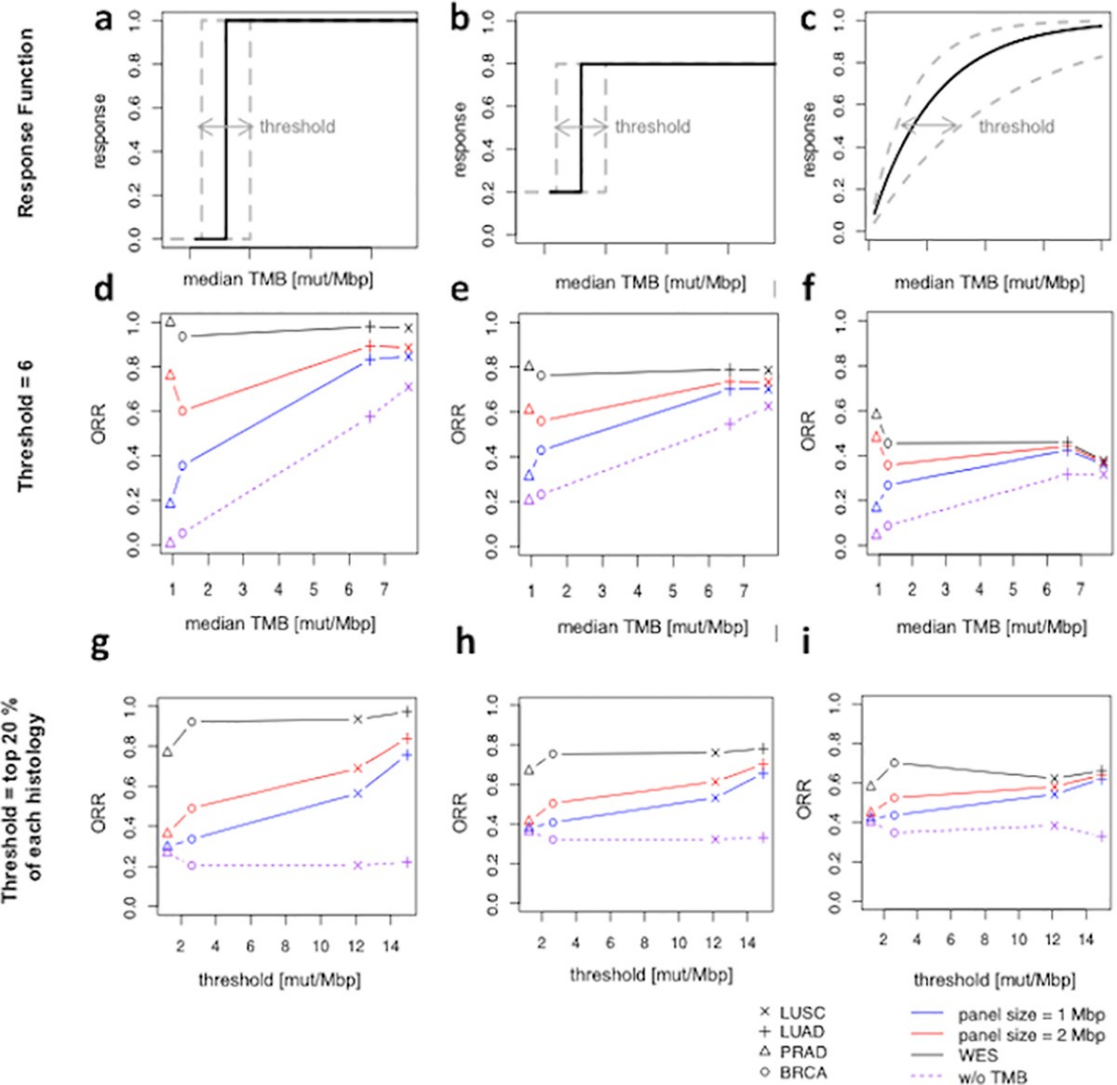
The model predicts the clinical outcome of a treatment group for different cancer types, thresholds, and panels. **a**-**c**, different response functions are assumed to predict the clinical outcome. Dotted lines are the schematic representations of each response function, when the threshold is set based on the top 20% of TMB distribution for each histology. **d**-**f**, histology and panel size impact the ORR for a fixed threshold. Smaller panels (higher noise content) are associated with poor clinical outcome. **g**-**i**, ORR when a threshold is selected based on the top 20% of the TMB distribution for each cancer type. PPV: positive predictive value.

We seek to elucidate how the inaccuracies arising from the use of panels as CDx devices can impact the likelihood of success in a clinical trial. To start, we model TMB as a pan-cancer predictor of response to immunotherapy treatment, where drug response is modeled as independent of histology (i.e. identical response function for all cancer types, see Methods and Fig 2A). Note that the choice of the specific response function in Fig 2A models TMB as an idealized perfect predictor of ORR that in this case converges to PPV (see Methods). Importantly, the pan-cancer predictive value of TMB would have been difficult to detected in wet-lab experiments because panel size affects the predictive performance of TMB in a histology dependent way. The observed drug-response in the high TMB group is significantly dependent on histology when using small panels (Fig 2D). Lower TMB in certain cancer types can exacerbate the overall poor clinical outcome of the entire selected cohort for immunotherapy treatment.

As discussed earlier, linear regression relates TMB measurements from a panel to WES differently for different panels and histologies and is an inappropriate tool to standardize TMB measurements. Consequently, we evaluated alternative potential TMB standardization approaches. One might argue that the response function (i.e. threshold setting) depends on the cancer type. Samstein *et al*. [2] suggested that using the top 20% of TMB distributions for each cancer type as threshold can standardize TMB measurements. Our model suggests that setting the threshold based on the top 20% of the entire population does not resolve the tissue dependency of clinical outcome, and both tissue type and panel size can affect ORR (Fig 2G). Conversely, our model implies that ORR is independent of histology if and only if WES (with minimal noise) is used (Fig 2D and 2G). Notably, assuming a perfect response function, a breast cancer cohort in which patients are selected to enroll in treatment using a panel of size 1 Mbp tends to demonstrate only a 35% chance of response compared to a 20% chance of response of a cohort in which TMB is not used as CDx. On the contrary, assuming WES is noiseless, the response with a WES based assay in the treatment group would be 100% (Fig 2D).

One might attempt to harmonize TMB measurements across different panels using alternative normalization methods (e.g. Z-score normalization, quantile normalization, etc.) [20]. However, it should be noted that any linear transformation of TMB distributions will lead to the same conclusion (since the pre-transformed TMB values that are above a certain quantile remain above that quantile after any linear transformation). Alternatively, the model suggests that designing panels to capture only non-cancer driver mutations and improved germline filtering can result in a superior performance (OPA); however, ORR of the selected population still continue to rely on panel size, cancer type, and the TMB threshold (S3 Fig). The dependency of ORR on histology persists but is alleviated if a response function predicts 80% ORR for patients with high TMB compared to 20% ORR for low TMB patients (Fig 2B, 2E and 2H). This observation inspired us to investigate clinical outcome using a more realistic response function.

Due to lack of adequate experimental data, obtaining an accurate response function that thoroughly captures all underlining biological features of the immune response is not trivial. Nevertheless, we pursued a mechanistic approach by first demonstrating that an incomplete gamma function can roughly encompass the underling biological machinery responsible for various stages of the immune response from gene expression to neoepitope presentation (see Methods). We followed a fitting strategy based on the available experimental data for NSCLC [21] to determine the shape and rate parameters. We reasoned that the shape parameter is associated with the number of epitopes required for immune cell activation. Consistent with the notion of immunodominance [22], our fitting approach resulted in a shape parameter of one. Following a tissue-agnostic assumption of the response function, we first estimated the clinical outcome of different cancer types, using the fitted response function (Fig 2C). Consistent with other discussed response functions, ORR varies significantly for different panels and tissue types (Fig 2F). Finally, these observations persist upon setting the rate parameter such that the top 20% of TMB population for each histology leads to 50% chance of response (the shape parameter remains unchanged, see Methods and Fig 2I). ORR of lung cancer patients for shape = 1, 2, and 3 for different panel sizes and thresholds are provided in S4B–S4D Fig.

Here we aimed to study the impact of the choice of response function by comparing the survival outcome given different response functions and how they can affect the clinical outcome as shown in Fig 2A–2C. Most studies focus on TMB high/low classification accuracy as a proxy for survival. This converges to the assumption that the response function is indeed what is depicted in Fig 2A (as well as Fig 2D and 2G). Note that assuming WES CDx could achieve 100% response rate is not justified. Therefore, we assessed the performance of different panels (and WES) assuming 80% response rate for high TMB patients as opposed to 20% response rate for low TMB patients (Fig 2B, 2E and 2H). Moreover, we utilized the empirical response data by Rizvi et al. [21] (S4A Fig) and obtained an empirical response function using a fitting strategy to demonstrate the importance of a priori knowledge of the response function (see Methods). In Fig 2B, 2E and 2H, we used a threshold of 6 as a choice to facilitate a graphical representation of the clinical outcome. However, the choice of the threshold can vary from cohort to cohort and therefore, we also provided a heatmap of the clinical outcome using empirically generated response functions (Rizvi et al. [21]) for different thresholds and different panel sizes (S4B–S4D Fig).

This model can also estimate how different factors (e.g. panel size) may influence the fraction of patient population stratified as potential responders when a targeted panel is employed as a CDx. Interestingly, the model suggests that at a constant threshold, smaller panels overestimate the number of potential responders (treatment population) which can jeopardize the likelihood of success of a clinical trial (e.g. a ~10% larger LUAD cohort may be chosen using a panel with L = 1 Mbp compared to WES, S5 Fig).

## Discussion

**Fig 3:**
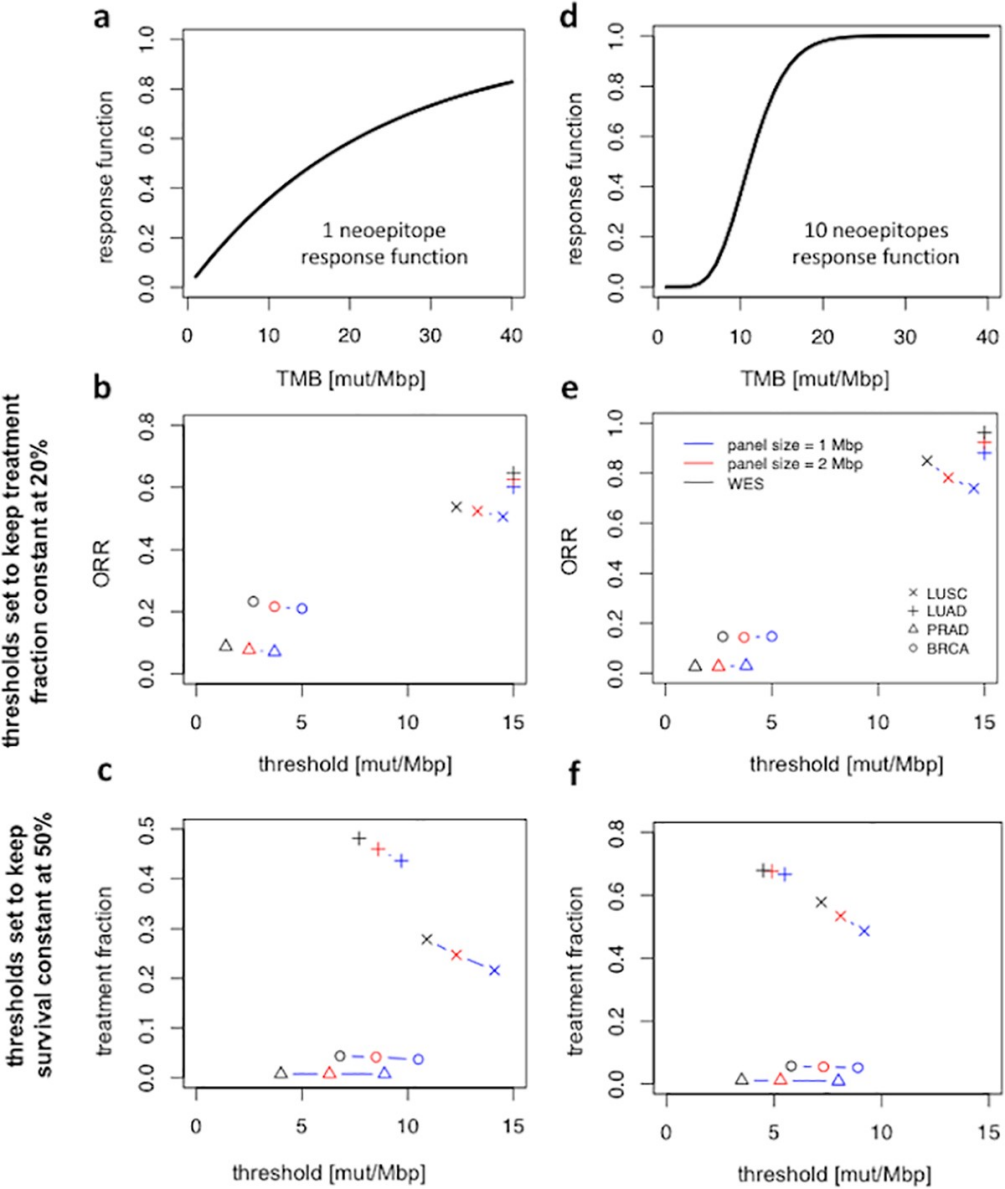
Proposed TMB harmonization strategy. TMB harmonization would only be possible if the exact response function is known and only when it is aimed to obtain identical fractions of treated patients or to obtain identical ORR in a given cohort but not both. **a** and **d** are two example response functions obtained using an incomplete gamma function which recognizes 1 and 10 neoepitopes, respectively. **b** and **e,** ORR when threshold is set to keep treatment fractions constant at 20%. **c** and **f**, treatment fractions for thresholds that keep ORR constant at 50% for the response functions shown in **a** and **b**.

This study conveys an important message tackling the main challenge in measuring TMB: how can one harmonize TMB across different panels, thresholds, and histologies? As discussed thoroughly throughout this study, TMB harmonization can only be achieved if the exact response function is known. Inspired by our model, one can obtain a threshold that results in either identical fractions of treated patients across different panels and histologies or identical ORR in a given cohort but not both (Fig 3). Thus, it is critical to clarify the scope of TMB harmonization when different panels or histologies are compared. One would expect that a small panel and an exome panel TMB thresholds are matched in such a way that the same percentage of the population is classified as “TMB high”. However, as shown in Fig 3B and 3E, one should expect a worse clinical outcome (as high as 20% reduction in ORR) if the TMB threshold is selected in such manner (Fig 3B and 3E). Alternatively, one could select the threshold such that a clinical trial in which a small panel is used achieves a similar ORR as an exome panel. However, this results in a smaller selected population for immunotherapy treatment some patients of which were true responders (Fig 3C and 3F). Nevertheless, a TMB threshold that leads to the same selected population and the same ORR between the two clinical trials does not exist.

Namely, given a known response function, our model can efficiently identify the proper threshold that stratifies the top 20% of patients as responders; however, the ORR varies depending on panel size and threshold. Likewise, when a threshold is aimed to yield a 50% ORR, the treatment fraction remains variable for different panels or histologies. Notably, WES entails ~10–20% larger treated population compared to a panel of size 1 Mbp depending on the response function.

In summary, we presented a mechanistic model that explains the association between panel size, histology, and TMB threshold with panel performance and survival outcome (S2P–S2R Fig). Our study suggests that TMB classification and threshold setting are only meaningful when all factors (i.e. noise characteristic of a panel due to varying panel sizes and TMB distribution per tumor type) are considered. This model can effectively be recruited to evaluate other potential TMB standardization approaches. Finally, the likelihood of a favorable clinical outcome can be predicted by our proposed model facilitating the design of future clinical trials [3]. Future studies based on larger cohorts can provide a more confident representation of the response function which assists us to achieve a more precise estimate of the influence of the aforementioned factors in predicting clinical outcome.

## Methods

### Model description

Assuming there exists a response function Ψ(*T*) defined as the probability of a patient with exact TMB (*T*) to respond to treatment, the probability of treatment response (the *hazard ratio* (HR)) of the entire selected patient cohort is

$$P_{response}(\tau ,\sigma )=\frac{1}{Z(\tau ,\sigma )}\int dT\Pi (T)\Psi (T)\Theta (T,\tau ,\sigma )$$ (2)

and the fraction of patients selected for treatment (market size) is

Z(τ,σ)=∫dTΠ(T)Θ(T,τ,σ) (3)

In these equations, Π(*T*) is the (cancer type dependent) distribution of mutation load in the intended use population. Θ(*T*,*τ*,*σ*) is a (noisy) biomarker model for a given CDx device and encodes the probability that a patient with true TMB (*T*) is selected for treatment based on a threshold *τ* and can be defined as

$$\Theta (T,\tau ,\sigma )=P(\tilde{t}/L>\tau )$$ (4)

where $\tilde{t}$ is the number of mutations recorded by a panel of size *L* and can be estimated as

$$\tilde{t}=Kt+N(0,\sigma )+Poiss(\lambda )$$ (5)

where *K* = *L*/*L*_0_ and *L*_0_ is the size of human exome (approximately 35.6 Mbp) such that *T* = *t*/*L*_0_. Eq (5), contains two noise terms: a centered gaussian noise source that represents the noise characteristic of a panel and is defined as

$$\sigma =C_{0}+\sqrt{Kt}$$ (6)

Note that *σ* is a function of panel size and mutational burden, and mutational burden is related to cancer type. Therefore, *P*_*response*_(*τ*,*σ*) in Eq (1) can also be written as a function of cancer type and panel size as *P*_*response*_(*τ*,*L*,*histology*). Moreover, we introduced a second Poisson noise term to recapitulate the biases due to cancer driver mutations, germline mutations, etc. *λ* and *C*_0_ are two tissue invariant constants on which together with Π(*T*) the model will be fitted. Here, *C*_0_ is a panel size independent noise source (such as germline subtraction noise), and *λ* is the average number of “cancer driver mutations” detected by the panel. We assume *C*_0_ and *λ* do not depend on the panel size and the tissue type since most commercial panels include cancer driver mutations regardless of the size of a panel. Such driver genes are biologically selected throughout the clonal evolution of cancer and thus, have a substantially higher probability of being observed on a targeted panel that is specifically designed to detect such variants rather than the passenger mutations that dominate TMB. Previous studies [23, 24] have modeled the panel intrinsic noise in measuring TMB by showing that the coefficient of variant of panel based TMB is inversely proportional to the square root of TMB and the panel size similar to this study. Moreover, J. Budczies et al. [24] have discussed a variety of confounders of panel based TMB measurements. Namely, the number of false negatives of germline mutation filtering, biological and technical panel based TMB error increase with the TMB level in a linear manner and thus result in constant relative errors.

Since $\tilde{t}$, the number of mutations recorded by a panel, must be an integer, the righthand side of Eq (5) is discretized and only $\tilde{t}>0$ values are considered. This is consistent with *in silico* and the observed TSO500 measurements as shown in Fig 1A–1C–(left corner). Assume a sample with true TMB *T* measured on a panel of size *L*. Then the expected number of non-synomymous mutations observed on the panel is $<\tilde{t}>=L\times T$. The statistics underlying this is a *Bernoulli* process [with p = *T* / 10^6^ per base] with the number $\tilde{t}$ “*successes*”. This allows to estimate the significance $\sigma =\sqrt{Lp(1-p)}\approx \sqrt{\tilde{t}}$ for small *p*. Again, using the normality assumption, we can conclude the 95% of measurement results are within two standard deviations $L\times T\pm 2\sqrt{L\times T}.$ Finally, we include *C*_0_ into Eq (6) to account for other sources of noise (e.g. germline subtraction noise).

It is worth noting that a noiseless device could be described as

$$\Theta (T,\tau ,\sigma =0)=\left\{ \begin{array}{c} 1\;if\;T\geq \tau \\ 0\;otherwise \end{array}\right.$$ (7)

Other quantities, such as sensitivity and specificity can be derived in a similar fashion.

### Model fitting

The distribution of tumor mutation load Π(*T*) for each tissue type can be estimated using TCGA data. Due to heterogenous mutational landscape of different tumor types, identifying an analytical pan-cancer probability density function for Π(*T*) is not trivial. Hence, without loss of generality, we fitted a kernel smoothing density function per cancer type.

Θ(*T*,*τ*,*σ*) includes various noise sources as a function of panel size and is also trained on TCGA data with two degrees of freedom (*λ* and *C*_0_). To assess the performance of the model, we first trained the model on two lung cancer subtypes (LUSC and LUAD) such that for any TMB measurements using WES the model simulates the TMB measurements of any panel of length *L*. The choice of *λ* and *C*_0_ did not dramatically influence the conclusions of this study for a range of parameters. Using a grid search, we chose *λ* and *C*_0_ to achieve a TMB distribution predicted by our model similar to TCGA i.e. Fig 1A and 1B. Specifically, *λ* = 1 and *C*_0_ = 0.5 showed a comparable concordance to the observed TMB distribution and thus were selected for this study.

### Response function

We studied three distinct response functions, but other response functions can easily be included for future analysis (Fig 2A–2C). In Fig 2D, and e, a sharp transition occurs at a selected tissue invariant threshold (e.g. 6 in Fig 2D and 2E) whereas the thresholds used in Fig 2G, and h are determined based on the top 20% for each cancer type (from TCGA). Next, we discussed the behavior of different panels using a more realistic response function (i.e. an incomplete gamma function).

*Corollary*. If a series of events occur according to a Poisson process with rate *λ*, the waiting time to the occurrence of the *n*th event, *T*_*n*_, follows a gamma distribution with the shape and rate parameters of *n* and *λ*.

Therefore, assuming that the immune response depends on the presentation of *n* neoepitope, the response function (*cumulative hazard function*) can be shown to follow a lower incomplete gamma function with the shape and rate parameters *n* and *λ*. We found the best fit by minimizing the ordinary least square (OLS) error of the response function and objective response as a function of the exact TMB reported by Rizvi *et al*. [21]. Optimization demonstrated that *n* = 1 minimizes the OLS error referring to the immunodominant neoepitope. We assumed an incomplete gamma function in Fig 2F with a shape parameter of 1 and rate = 0.044 (obtained by OLS minimization, S5A Fig). Likewise, in Fig 2I, the rate parameter was determined such that 50% of ORR occurs at the top 20% per histology (assuming shape = 1).

### Whole exome and TSO500 TMB workflows

We followed the protocol described in [25] for all TMB calculations including alignment, variant calling, removing germline variants, mutational load measurement, etc.

## Supporting information

S1 Table TMB data generated for a) WES samples form TCGA, b) *in silico* TSO500, c) WES and TSO500 sequenced in house for this study for an independent NSCLC cohort.
(XLS)

S1 Fig The model predicts the noise behavior of different panels. **a,** noise characteristic of different panels for different TMB values. Lowess smoothing used to illustrate the noise content of different panels. FMI: Foundation Medicine panel. TSO500 (model) and FMI (model) are generated using the mathematical model described in this study with panel size length as the input. **b,** contribution of different sources of noise to the regression offset for different panels. **c,** tissue dependency of regression coefficients between two panels (1 Mbp vs. 2 Mbp).
(TIF)

S2 Fig Classification accuracy for different panels, given different thresholds for different histologies. **a-c,** negative percent agreement (NPA). **d-f,** positive percent agreement (PPA). **g-i,** overall percent agreement (OPA). **j-l,** positive predictive value (PPV). **m-o,** negative predictive value (NPV) for 3 thresholds (6,8, and 10). LUSC: lung squamous cell carcinoma, LUAD: lung adenocarcinoma, PRAD: prostate adenocarcinoma, BRCA: breast invasive carcinoma. **p-r,** ORR given a tissue agnostic response function (Fig 2C).
(TIF)

S3 Fig The model predicts the classification performance of different panels for different cancer types for TMB measurements using a panel empty of cancer driver mutations and germline variants. **a**, removing germline variants and cancer driver mutations result in a more efficient panel (smaller panel with identical performance); however, this approach can not effectively reduce the intrinsic noisy behavior of panels and classification performance remains to depend on panel size (b-d). Contour colors show the density of data points per contour.
(TIF)

S4 Fig ORR for lung cancer patients. **a,** data points represent response status for each patient given the exact recorded TMB by WES (Rizvi *et al*.) and the response function is obtained by fitting an inverse gamma function with the shape parameter = 1. **b-d,** heatmaps of ORR for lung cancer patients obtained using the response function in **a**, for different shape parameters (number of neoepitopes) for a range of thresholds and panels.
(TIF)

S5 Fig Fraction of patients selected for treatment using different panels. smaller panels overestimate the market size.
(TIF)
