# Supplementary figures and images for: The interplay between cancer type, panel size and tumor mutational burden threshold in patient selection for cancer immunotherapy
Source: PLoS Comput Biol. 2020 Nov 9;16(11):e1008332. doi: 10.1371/journal.pcbi.1008332 (PMC7676656; doi:10.1371/journal.pcbi.1008332)

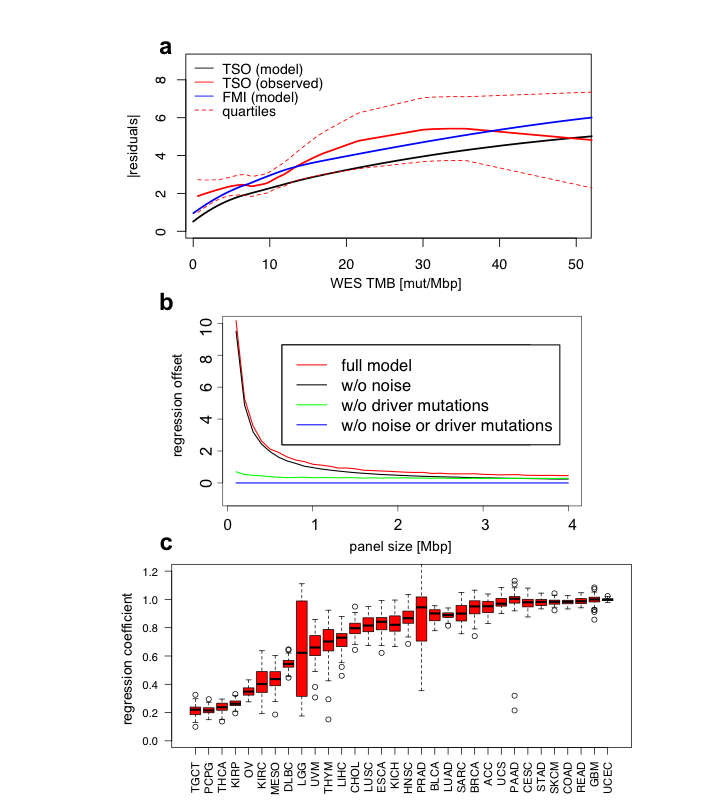

Supplement: S1 Fig — a, noise characteristic of different panels for different TMB values. Lowess smoothing used to illustrate the noise content of different panels. FMI: Foundation Medicine panel. TSO500 (model) and FMI (model) are generated using the mathematical model described in this study with panel size length as the input. b, contribution of different sources of noise to the regression offset for different panels. c, tissue dependency of regression coefficients between two panels (1 Mbp vs. 2 Mbp). (TIF) [file pcbi.1008332.s002.tif]

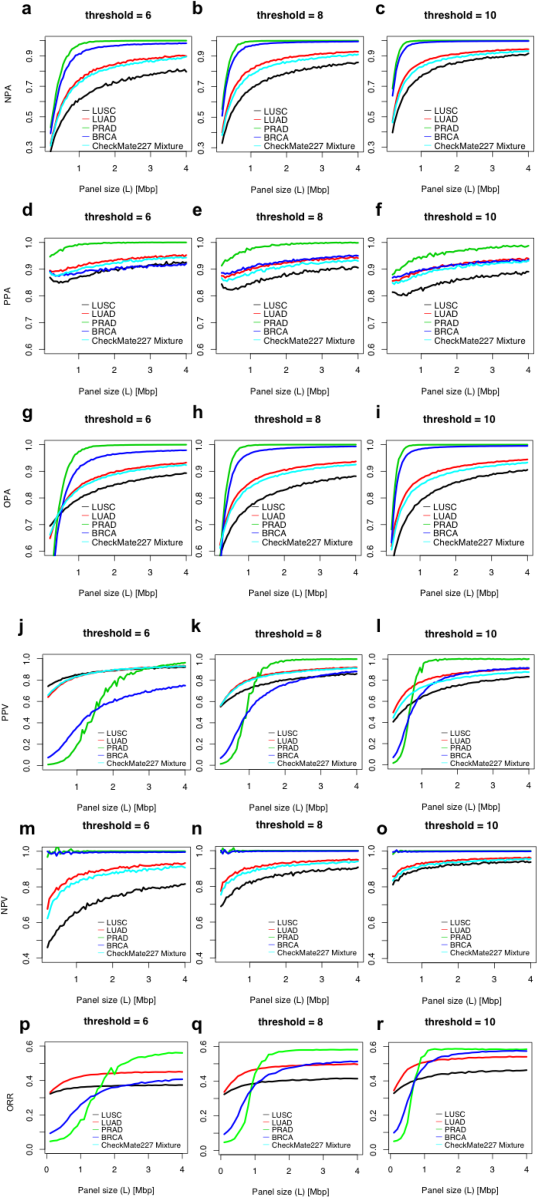

Supplement: S2 Fig — a-c, negative percent agreement (NPA). d-f, positive percent agreement (PPA). g-i, overall percent agreement (OPA). j-l, positive predictive value (PPV). m-o, negative predictive value (NPV) for 3 thresholds (6,8, and 10). LUSC: lung squamous cell carcinoma, LUAD: lung adenocarcinoma, PRAD: prostate adenocarcinoma, BRCA: breast invasive carcinoma. p-r, ORR given a tissue agnostic response function (Fig 2C). (TIF) [file pcbi.1008332.s003.tif]

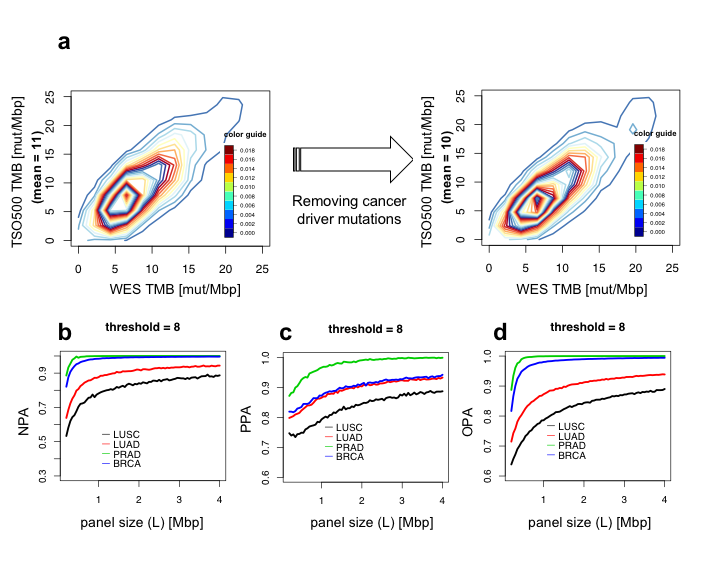

Supplement: S3 Fig — a, removing germline variants and cancer driver mutations result in a more efficient panel (smaller panel with identical performance); however, this approach can not effectively reduce the intrinsic noisy behavior of panels and classification performance remains to depend on panel size (b-d). Contour colors show the density of data points per contour. (TIF) [file pcbi.1008332.s004.tif]

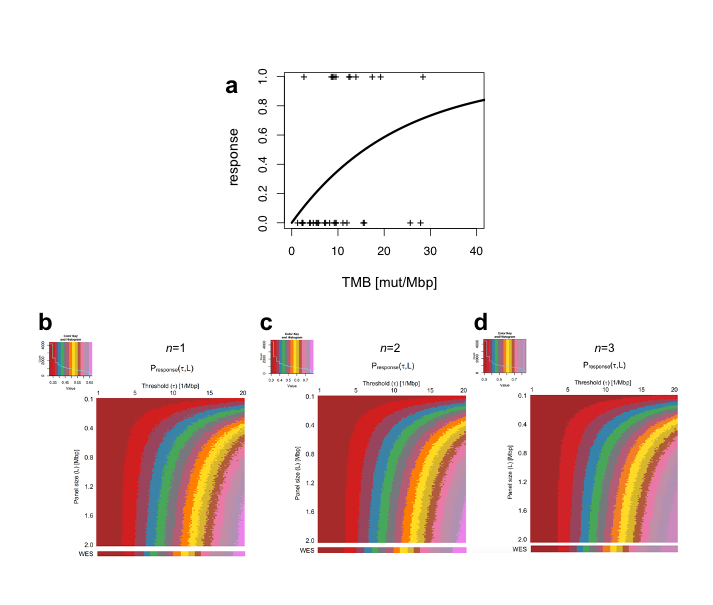

Supplement: S4 Fig — a, data points represent response status for each patient given the exact recorded TMB by WES (Rizvi et al.) and the response function is obtained by fitting an inverse gamma function with the shape parameter = 1. b-d, heatmaps of ORR for lung cancer patients obtained using the response function in a, for different shape parameters (number of neoepitopes) for a range of thresholds and panels. (TIF) [file pcbi.1008332.s005.tif]

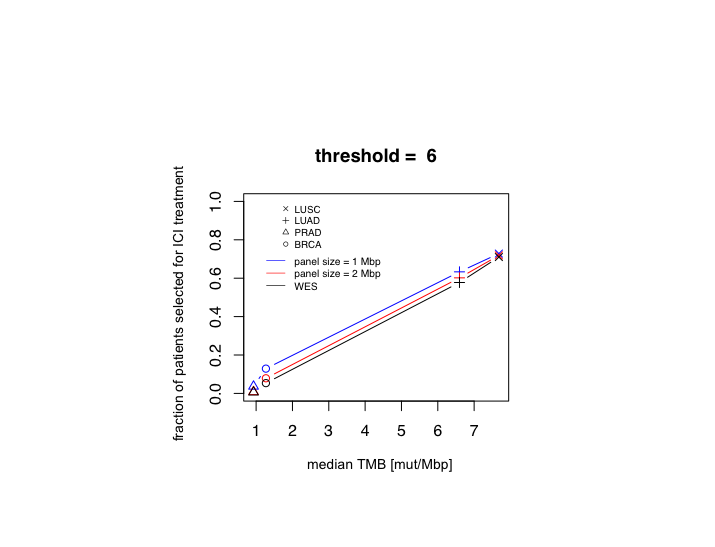

Supplement: S5 Fig — smaller panels overestimate the market size. (TIF) [file pcbi.1008332.s006.tif]
